# Who polarizes Twitter? Ideological polarization, partisan groups and strategic networked campaigning on Twitter during the 2017 and 2021 German Federal elections 'Bundestagswahlen'

**DOI:** 10.1007/s13278-022-00958-w

**Published:** 2022-10-11

**Authors:** Philipp Darius

**Affiliations:** Center for Digital Governance, Hertie School, Berlin, Germany

**Keywords:** Political Communication, Digital Campaigning, Twitter Networks, Hashtag Debates, Online Polarization

## Abstract

Political campaign activities are increasingly digital. A crucial part of digital campaigning is communication efforts on social media platforms. As a forum for political discourse and political communication, parties and candidates on Twitter share public messages and aim to attract media attention and persuade voters. Party or prominent candidate hashtags are a central element of the campaign communication strategy since journalists and citizens search for these hashtags to follow the current debate concerning the hashed party or political candidate. Political elites and partisans use social media strategically, e.g., to link their messages to a broader debate, increase the visibility of messages, criticize other parties, or take over their hashtags (hashjacking). This study investigates the cases of the most recent 2017 and 2021 German federal elections called 'Bundestagswahlen'. The investigation (1) identifies communities of partisans in retweet networks in order to analyze the polarization of the most prominent hashtags of parties, 2) assesses the political behavior by partisan groups that amplify messages by political elites in these party networks, and 3) examines the polarization and strategic behavior of the identified partisan groups in the broader election hashtag debates using #BTW17 and #BTW21 as the prominent hashtags of the 2017 and 2021 elections. While in 2017, the far-right party 'Alternative für Deutschland' (AfD) and its partisans are in an isolated community, in 2021, they are part of the same community as the official party accounts of established conservative and liberal parties. This broader polarization may indicate changes in the political ideology of these actors. While the overall activity of political elites and partisans increased between 2017 and 2021, AfD politicians and partisans are more likely to use other party hashtags, which resulted in the polarization of the observed parts of the German political twitter sphere. While in 2017, the AfD polarized German Twitter, 2021 shows a broader division along the classical left–right divide.

## Introduction

Social media communication as digital campaigning is essential to political election campaigns (Gibson and Römmele 2004; Gibson et al. 2014; Lilleker et al. 2017). The COVID-19 pandemic further increased the importance of digital campaign tools, e.g., for the 2021 German federal elections, the 'Bundestagswahlen 2021'. For political candidates, politicians and parties, journalists, party supporters, and politically interested citizens, Twitter is an essential platform for public debates. These public debates on Twitter organize around hashtags that link users' tweets to a particular topic. The acronyms of the German word for the federal elections (Bundestagswahlen) have been among the most frequently used hashtags^1^ (#BTW17 and #BTW21) to connect campaign tweets to the general election issue during the election cycles.

While prior work monitored the frequency of political party and candidate hashtags in German election campaigns on Twitter, previous analyses do not account for the strategic use of hashtags regarding political opponents (Stier et al. 2018b). Several scholars named this strategy hashtag hijacking (Hadgu et al. 2013; Van Dam and Tan 2016, Xanthopoulos et al. 2016) or, in short, 'hashjacking' (Bode et al. 2015; Darius and Stephany 2019). In the German context, prior work showed that the German far-right party 'Alternative für Deutschland' (AfD) used hashjacking as a political communication strategy (Darius and Stephany 2019). Additionally, politicians and partisans shared vaccination-skeptic messages during the COVID-19 pandemic and used hashtags strategically to influence the broader debate on the pandemic (Darius and Stephany 2022). This study extends prior work by investigating the strategic hashtag use by political elites and partisans during the final phase of the 2017 and 2021 German federal election campaigns. Election campaigning comprises a unique opportunity to observe political communication efforts by parties and candidates. Correspondingly, the study examines strategic hashtag use of political party hashtags and compares the polarization of these hashtag discourses during the 2017 and 2021 election campaigns.

The study proceeds as follows: the background (2) section briefly outlines different historical phases of political campaigning and the role of the media (2.1). After that, it discusses the use and functions of digital campaigning methods focusing on political communication on social media (2.2). Then it discusses online polarization and strategic political behavior online, such as hashjacking (strategic hashtag use of other political parties' hashtags), which comprises a strategy predominantly used by right-wing populists/far-right actors (2.3). Section (3) formulates the research hypotheses based on existing literature and prior work. Section 4 introduces the research design based on data collection (4.1), network approach (4.2), and explanation of the measures (4.3). Successively, Sect. (5) presents the analysis and results, followed by the discussion Sect. (5) that reviews the findings (5.1), limitations of the study (5.2), and concludes with a reflection on the broader meanings of the findings for political campaigns and democracy.

## Background

Studying online polarization and strategic communication as digital campaigning speaks to political science, media studies, network science, and interdisciplinary social media research. While this section introduces literature on Twitter as a campaigning tool and forum for public debate and reflects on strategic political behavior on Twitter, it also aims to provide an interdisciplinary readership with a short introduction to the development of political campaigning and the media. Correspondingly, this section first introduces a short history of political campaigning and then reviews the most relevant literature on the strategic use of social media in political campaigning and communication.

### Political campaigning and the media as a process

In order to grasp changes in digital political campaigning, it is crucial to contextualize the historical development and different phases of political campaigning in the past two centuries. Most of the existing literature distinguishes three or four eras of political campaigning that correspond with broader technological and societal developments (Strömbäck 2008; Hjarvard 2013; Esser and Strömbäck 2014; Couldry and Hepp 2017; Hepp 2019). It is common to differentiate between premodern, modern, and postmodern periods of political campaigning and their typical campaign practices (Schmitt-Beck and Farrell 2002; Norris 2004). More recently, scholars argued that campaigning moved into a fourth phase due to the influence of digitalization on the media and politics (Strömbäck 2008; Blumler 2016,; Magin et al. 2017; Römmele and Schneidmesser 2016; Römmele and Gibson 2020). The Democratic Obama campaign in 2008 is often seen as an early exemplary case, laying out strategies of how to implement web-based communication and "big data" in a political campaign (Gueorguieva 2006; Lilleker and Jackson 2013; Gerodimos and Justinussen 2015).

The catalyzation of trends fueled by technological change distinguishes the fourth era from prior eras, leading to a new form of data-driven campaigning (Römmele and Gibson 2020). With the growing adoption of social media platforms and the Internet, web-based or digital political campaigning has spread globally (Gibson et al. 2014; Dimitrova and Matthes 2018). Due to the wide use of social media in politics and the related power of platforms as information gatekeepers, social platform architecture and governance become crucial factors for the quality and legitimacy of democracy (Gillespie 2018; Cowls et al. 2022; Stockmann 2022). This increased importance of digital technologies and data analysis in campaign operations and organization is reflected in the transition of political communication from mass media-based to a more direct, interactive, and networked type of communication with the electorate, targeting of campaign messages, and an increasingly international dimension of political campaigns with interferences by foreign actors. Moreover, a qualitative separation of digital and data-driven campaigns may occur into a rational-scientific approach on the one hand and an emotionalized subversive approach to campaigning on the other that may benefit populist parties (Römmele and Gibson 2020). Social media provides a platform for public debate and communication beyond traditional media like print, television, or the radio. The following section will discuss Twitter's role as a forum for political debate and a political communication tool in election campaigns.

### Twitter as a political communication tool and forum for public discourse

From the beginning of social media use in politics, academics have asked whether it would enhance direct communication between politicians, journalists, and citizens. This direct communication could indicate a more participative or, to quote Habermas, a more "public sphere-like" democratic space (Habermas 1991; Ferree et al. 2002; Dahlgren 2005; Colleoni 2014; Ekman and Widholm 2015; Rau und Stier 2019).^2^ In several studies, there was no consistent indication of increased communication between political 'elites' and citizens, but politicians and journalists communicate primarily with each other in public (Grant et al. 2010; Verweij 2012; Nielsen and Vaccari 2013; Oelsner and Heimrich 2015; Jensen 2017). Within these online spaces, candidates, political representatives, and parties communicate with the public, and journalists and citizens interact with these messages (Gibson et al., 2014). Concerning the capacities for campaigning and the central questions of which target groups political communication reaches, social media differ significantly due to mediation effects based on their varying structures, communication mechanisms, and user audiences (Bossetta et al. 2018, Stier et al. 2018b, Bronstein et al. 2018). In Germany, for instance, Facebook reaches a much broader demographic group than Twitter^3^ and parties may leverage social media to reach young voters (Copeland and Römmele, 2009). On Twitter, however, elite actors such as politicians react to trends such as rising hashtag debates and aim to influence media reporting, meaning that journalists cover their political messages, which has multiplier effects in reaching the public (Larsson and Kalsnes, 2014; Kreiss 2018). In general, studies question whether social media lead to interactions between voters and politicians (Graham et al. 2013; Oelsner and Heimrich 2015; Caton et al. 2015). During campaign periods, politicians, such as members of parliament in Germany, tend to use Twitter more actively and differently to non-campaigning times. For instance, they refer more often to the broader election topics or hashtags instead of sharing content from their personal lives (Nuernbergk and Conrad 2016).

With regards to election campaigns, social media play a multifaceted role. Social media as digital tools for political elites affect political campaigning practices mainly by their four main functions (1) organizational structures and work routines, (2) presence in online information spaces, (3) support in resource collection and allocation, and (4) symbolic uses in the sense of political marketing (Jungherr 2016). Symbolic uses and presence in online information have been the focus of scholars working on populism, extremism, and media research. For extreme parties and their political narratives, social media offer additional channels for political communication in which extreme political actors do not need to follow the values and norms of traditional mass media and are thus able to spread their respective ideologies (Engesser et al. 2017). The ideology of right-wing populist parties builds on the rhetoric construction of (1) anti-elitism/establishment, (2) anti-migration, and (3) anti-Muslim stances. Notably, these three pillars of right-wing populist rhetoric and policies polarize voters against something and, in particular, against certain groups of people (Mudde 2004; Mudde and Kaltwasser 2013). Social platforms and messengers are desirable for right-wing populist and radical-right parties as political challengers who often have a hostile attitude toward established media and sometimes limited access to traditional media channels (Engesser et al. 2017; Jungherr et al. 2019; Koc-Michalska and Klinger 2021). Thus, right-wing populist actors and movements have benefited disproportionately from the emergence of social media since they can circumvent traditional media and communicate directly to their target audiences (Stier et al. 2018b; Jacobs 2018). Besides, direct contact with political actors and the represented ideologies enables the self-socialization of citizens into right-wing populist beliefs and worldviews (Krämer 2017; Schumann et al. 2021).

Furthermore, social media also provides an opportunity for top-down leadership claims for populist parties and politicians. Social media provided additional channels for communication with and between political elites, partisans, and the electorate. Hashjacking of political adversaries and the strategic hashtags use of broader discourses increase the representation of populist messages on social media (Darius and Stephany 2019, 2022). What remains unclear is whether strategic hashtag use and hashjacking also increase online polarization during campaign times.

### Online Polarization as a Strategy?

There has been conflicting evidence on the relationships between social media and socio-political polarization (Garimella and Weber 2017; Bail 2018, 2021). It is yet unclear whether social media (1) might reduce polarization by enabling access to a more diverse set of information and news (Stier et al. 2021) or (2) might increase or accelerate polarization tendencies by algorithmic enforcement of opinions, e.g., by the formation of so-called echo-chambers, or (3) whether online polarization on social media solely reflects differences in the social and political (offline) world.^4^ Even the assumption that exposure to a more diverse set of information and political views may reduce polarization is questionable or even counterfactual (Bail et al. 2018). Besides this unclarity about interpretations, various definitions of offline polarization hamper academic consensus (Bramson et al. 2017; Tucker et al. 2018). Regarding polarization, social media provide a chance to analyze political behavior by elites and partisans (Gil de Zúñiga et al. 2020). For instance, individuals self-sort into ideologically aligned communities by retweeting their behavior when using political party hashtags (Conover 2011; Conover 2012). This sorting happens because most users retweet in support of messages, especially regarding political hashtags and topics (Metaxas et al. 2015). Consequently, strategic hashtag use by politicians and users' retweeting behavior may result in polarized political hashtag debates in which communities reflect camps with contrasting political ideologies.

Hashtags enable Twitter users to interact in so-called ad-hoc publics outside their follower networks^5^ and link their tweets to a broader conversation (Bruns and Burgess 2011). Consequently, hashtags allow for ad-hoc (political) debate on Twitter and are used frequently by politicians and journalists (Enli and Simonsen 2018). Political elites and partisan groups employ strategic behavior such as retweeting or hashjacking (using political opponents' hashtags) to influence these public debates. Usually, elite actors such as politicians or social media influencers issue messages using hashtags strategically, and partisans amplify their messages by sometimes excessive retweeting. These strategic expressions may increase online polarization caused by political elites and partisans' self-sorting into ideological camps (Conover 2011; Garimella and Weber 2017).

Political elites strategically use party and campaign hashtags to increase their visibility and support their party in election campaigns. In contrast to traditional political communication via mass media, this constitutes a new form of so-called networked campaigning. Networked campaigning is characterized by the increasing importance of networked communication logic and logic of connective action (Bennett and Segerberg 2013; Klinger and Svensson 2015). These new logics result in equally new forms of strategic political communication behavior. Two forms of strategic political online behavior on Twitter that political elites and partisan groups frequently exercise are 1) "retweeting" (amplifying someone else's message) and 2) "hashjacking" (using hashtags of political opponents) as a particular form of hashtag use to take over the hashtag of political opponents (Bode et al. 2015). Regarding retweeting, in political contexts, many users only retweet messages they support, which results in ideologically aligned groups and polarized retweet networks (Conover et al. 2011). These partisan groups coordinate to use political opponents' hashtags, leading to spontaneous jumps in the polarity of hashtags or even polarizing hashtags in the long term (Hadgu et al. 2013; Weber et al. 2013). In the case of Germany, hashjacking was used strategically by the far-right party AfD as a polarization strategy (Darius and Stephany 2019) and a disinformation strategy during the COVID-19 pandemic (Darius and Stephany 2022). In practice, hashtag hijacking can be challenging to distinguish from strategic hashtag use, which connects individual messages to broader political debates. In the German case, however, far-right actors were much more likely to use other party hashtags, e.g., to hashjack, or link to broader COVID-19-related debates than partisans of other parties, which indicated a coordinated and strategic use by AfD politicians amplified by partisans (Darius and Stephany 2022). This isolation of the far-right partisans differs from other partisans or party supporters in one large community with other parties and journalists. Beyond national politics, partisan groups, such as far-right partisans, may also coordinate transnationally with the aim of hashjacking hashtags of social movements such as #MeToo (Sorce 2018; Knüpfer et al. 2020).

Regarding social movements, Twitter may also facilitate the spread of conspiracy narratives and enable the mobilization of disinformed social movements, e.g., during the COVID-19 pandemic (Darius and Urquhart 2021). While political elites, such as official politicians or political influencers, create content, partisans drive the dynamic by amplifying messages. This mechanism lies at the center of the analysis of strategic hashtag use and provides a fundament for formulating the research questions in the following section.

## Research questions

While prior work investigated the role of strategic hashtag use and 'hashjacking' as a strategy used predominantly by far-right actors in the German Twittersphere, this study explores the use of party, chancellor candidate, and broader election debate hashtags during the election campaigns. During campaign times, political debates intensify, which may result in hashtag debates that are more polarized in contrast to periods outside election cycles. Besides, political elites, such as parties, members of parliament or election candidates, and political partisans have higher incentives to behave actively on social media and criticize political adversaries for gaining media attention or persuading undecided voters. Revisiting prior work on 'hashjacking' (Darius and Stephany 2019; 2022) and Twitter use in (German) political campaigns (Stier et al. 2018a) and based on the presented literature on 'hashjacking' and strategic hashtag use, the study formulates the following research questions. It is worth noting that using a party hashtag when politically opposed is not automatically hashjacking. However, messages are often issued strategically by politicians and political influencers. Thus, only if there is a significant degree of coordination, as expressed in a higher presence and likelihood of partisans to use other parties' hashtags, should this be understood as hashjacking.

- (RQ1): Did AfD partisans aim to hashjack other parties' hashtags during the election campaigns in 2017 and 2021?
- (RQ2): Was the average activity of partisan communities around the party and election hashtags higher in 2021 than during the 2017 federal election campaigns?
- (RQ3a): Do AfD partisans appear as an isolated community in the #BTW17 retweet networks?
- (RQ3b): Do AfD partisans appear as an isolated community in the #BTW21 retweet network?
- (RQ4): Are right-wing partisans of the AfD more likely to engage in the macro-debate #BTW17 and #BTW21 hashtags than partisans of other parties?

## Research Design

**Fig. 1:**
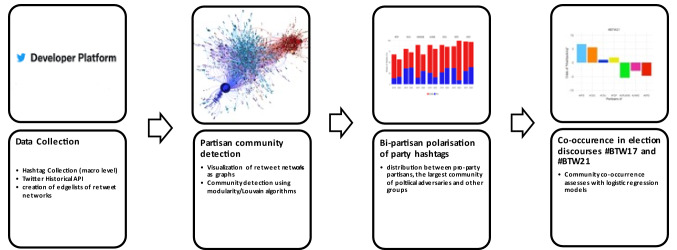
Pipeline of the analytical approach to assess the polarization of hashtags within and between hashtag discourses

This section elaborates on the data collection (4.1), introduces the network approach and community detection of partisan groups as an assessment of polarization (4.2), and presents the measurement approach (4.3) to further examine online polarization and strategic political online behavior during the 2017 and 2021 German federal election campaigns. Figure 1 illustrates the pipeline of the analysis.

### Data collection

The study collects Twitter data by accessing Twitter's application programming interface (API) for academics.^6^ Twitter's historical API access allows the retrospective collection of user timelines or tweets of hashtags. For two reasons, hashtags are the macro-level selection criteria (Weller et al. 2013). First, party hashtags are important campaign goals used by politicians, supporters, journalists, and the main party accounts. Secondly, journalists and citizens might use Twitter hashtags to inform themselves about the latest news on the party. This information function may consciously or unconsciously serve as an indicator of public support for that party. This online debate is not representative but may have significant consequences, e.g., influencing media reporting. Consequently, the collection focuses on party hashtags for all parties that entered the federal parliament (#AfD, #CDU, #CSU, #FDP, #GRUENE, #LINKE, #SPD) plus candidate hashtags as the last names of the three top candidates (#Baerbock, #Laschet, #Scholz). Additionally, the study analyses the broader debates on the elections represented by the most used hashtags regarding the Federal elections in 2017 and 2021 are #BTW17 and #BTW21 as the acronyms of the German word 'Bundestagswahlen.'

The observation period is the final week before the elections in 2017 and 2021, in which we expect intensified campaigning efforts by parties, candidates, and supporters. The study first identifies partisan communities and hashjacking efforts using party hashtags during the two observation periods and, second, investigates the role of these partisan groups within the broader political debate on the elections represented by the #BTW17 and #BTW21.

### Network approach for the detection of partisan communities

Political networks may represent many different relations, such as parliamentary co-sponsorship (Fowler 2006), coalition formation (Maulana et al. 2022), organizational ties (Hafner-Burton et al. 2009), or political news diffusion on social platforms (Grinberg et al. 2016). In social network analysis, nodes represent individuals, and edges indicate relations or interactions. When aggregated, this relational data enables the graphical visualization and statistical analysis of the structure of networks (Wassermann and Faust 1994; Carrington et al. 2005; Scott 2017). A network approach is favorable for the structural analysis of social media since communication on most platforms is networked by design. On Twitter, for instance, by linking to other users via @mentions, retweeting, or linking messages to a topic using hashtags (#). Retweeting creates a link (edge) between two accounts (nodes), constituting a network. These retweet networks often cluster into multiple communities, and for political hashtags, these communities may have different political party affiliations or ideologies (Conover 2012). Thus, retweet networks provide the chance to assess ideological alignment and opinion leaders within communities where people self-sort by their retweeting behavior (Conover 2012; Bruns et al. 2016). This self-sorting into different ideologically aligned communities occurs because most users retweet in support of messages they ideologically agree with (Boyd et al. 2010; Metaxas et al. 2015). Moreover, users adopt retweets quicker than using hashtags in individual tweets (Oliveira et al. 2021). Therefore, the analysis focuses on the retweeting networks of the chosen hashtags to identify partisan communities and assess the polarization of the political debates on Twitter.

The analysis assumes that the network consists of two major partisan clusters. The more significant the proportion of accounts in these clusters, the more polarized is the network. In prior work on the German context, partisans of the far-right AfD constituted an isolated community in several political party hashtags (Darius and Stephany 2019) and Covid-19-related hashtags (Darius and Stephany, 2022) retweet networks. In contrast, partisans and politicians of all other parties gather in a large community with journalists and media outlets. Consequently, AfD partisans, as supporters of a reasonably new anti-establishment party, have polarized the political discourse on Twitter as represented by retweet networks of common hashtags.

The analysis builds on the networked structure and the visualization of retweet networks in Gephi using the Force2 layout algorithm (Bastian et al. 2009; Jacomy et al. 2014). In the first step of the analysis, the modularity-based (community detection) algorithm assigns the nodes to different communities based on the structural properties of the network graph (Newman 2006; Blondel et al. 2008; Fortunato 2010). Being retweeted is highly unequally distributed (Bild et al. 2015). Therefore, a qualitative content analysis of the 30 most retweeted accounts in each party network makes sense of the clustering (White and Marsh 2006; Mayring 2014; Krippendorff 2018). Tables 2 and 3 illustrate an example of the analysis of the 30 most retweeted accounts in #BTW17 and #BTW21. To account for the skewed distribution of being retweeted, the study uses a log-transformation that works for most social network data and also produces acceptable results for social media data (Broido and Clauset 2019).

Partisan communities often center around official party accounts such as @AfD or @dieLinke (or the other party accounts). In prior studies, a closely connected far-right partisan community, whose activity was much higher than that of other communities, formed around official AfD accounts and amplified their political messages by retweeting (Darius and Stephany 2019). This pro-/contra-poformer party leader and chancellor candidate Arlarization of each party retweet network and assigned community memberships by the Louvain algorithm enables partisan groups' identification.^7^ In a further step, the analysis assesses the occurrence of these groups in the broader debates (here #BTW17 and #BTW21).

### Measurement of polarization and strategic hashtag use

The investigation of the research questions builds on a network approach that enables the visualization and analysis of the structure and identifies partisan communities in the party retweet networks via the Louvain community detection algorithm. The study uses the terms' community,' and 'cluster' interchangeably since communities of politicians and partisans appear as clusters in the network structure. In party hashtags, the proportion of AfD partisans to the pro-party community indicates the extent of hashjacking (RQ1). The analysis measures the average activity of partisan groups (RQ2) by the weighted outdegree retweet network of nodes in the pro-party partisan community within each party hashtag. The weighted outdegree, also accounting for the number of reciprocated edges, signifies the number of times an account retweeted another account. In order to examine (RQ3a) and (RQ3b), the study assesses the assumed isolation of the AfD partisan cluster in two steps. At first, the investigation of the retweet network topology indicates the networks' potential polarization between partisan communities, and second, a qualitative content analysis of the most retweeted accounts (with the highest indegree) indicates the political ideology of the communities.

The analysis proceeds with logistic regression models to test each partisan group's binary likelihood of co-occurrence in one of the two main clusters on the broader #BTW17 and #BTW21 debates. The response (independent variable) of being in one of the two largest communities in #BTW17 and #BTW21 is binary since most nodes are assigned to these two communities (see Table 1).

$$y_{i} = \left\{ {\begin{array}{*{20}l} {1,} \hfill & {{\text{if}}\;{\text{the}}\;{\text{ith}}\;{\text{node}}\;{\text{is}}\;{\text{co - occurring}}\;{\text{in}}\;{\text{the}}\;{\text{right - wing}}\;{\text{partisan}}\;{\text{cluster}}} \hfill \\ {0,} \hfill & {{\text{otherwise}}} \hfill \\ \end{array} } \right.$$

The realization of ${Y}_{i}$ (community membership in #BTW17 and #BTW21 retweet networks) ${y}_{i}$ is random and binary (0,1) with the probabilities ${\pi }_{i}$ and ${\pi }_{i}-1$. Assuming no ideological sorting, observations within each partisan group should have the same probability of co-occurring in the right-wing partisan community in #BTW17 and #BTW21. The distribution of ${Y}_{i}$ is binomial with the parameters ${\pi }_{i}$ and ${n}_{i}$; ${Y}_{i} \sim B({n}_{i}, {\pi }_{i})$.

The log-odds are calculated as: ${\eta }_{i}=logit\left({\pi }_{i}\right)=\mathrm{log}\frac{{\pi }_{i}}{1-{\pi }_{i}}$ and the odds for co-occurring in the partisan cluster (in the party retweet networks) and the right-wing partisan community in #BTW17 and #BTW21.

The logistic regression models assess cross-cluster homophily between partisan clusters and an identified right-wing community in the #BTW17 and #BTW21 retweet networks (RQ4). The resulting odds as outputs of the logistic regression models indicate how likely partisan groups from the party networks co-occur in the more extensive retweet networks of #BTW17 and #BTW21. A high likelihood of partisan co-occurrence in clusters of the broader electoral debate indicates the ideological closeness of these groups. Similar directions of the odds indicate membership in the same significant clusters. If partisans of one party are isolated in one community, as hypothesized for AfD partisans in (RQ3a) and (RQ3b), they should have the opposite odds direction as all other parties.

## Results

This section presents the results of the analysis. The first part compares German political party hashtags polarization between the two election periods in 2017 and 2021. The community detection identifies a pro-partisan cluster and a contra cluster as the largest community with highly retweeted members of other parties. Then, the analysis compares the activity of different partisan groups. After that, the focus lies on the structural assessment of the large-scale hashtags #BTW17 and #BTW21 and investigates the likelihood of (strategic) hashtag use of different partisan communities. The main finding is a differently polarized electoral debate on Twitter between 2017 and 2021. While the far-right party AfD was a segregated community in 2017, in 2021, the AfD is in the same cluster as the center-right, conservative parties (CDU and CSU) or the liberal party (FDP). Section (6) further discusses this central finding and the findings regarding the individual hypotheses.

### Partisan polarization of party hashtags

**Fig. 2:**
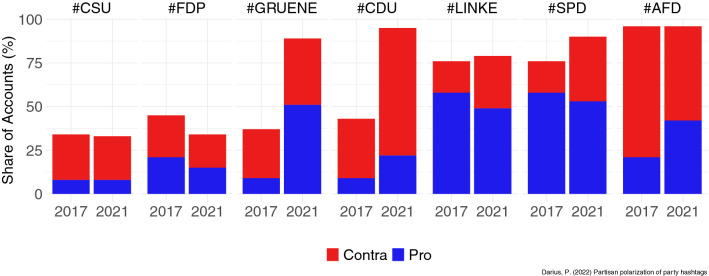
Column chart of pro-partisans for each party and contra-communities as members of the largest community consisting of other partisans. A qualitative content analysis of the most retweeted accounts identifies partisanship and ideological differences between communities. The contra-communities for all parties except the AfD consist of AfD partisans, whereas the contra community in the AfD network consists of other parties' partisans and official accounts as well as media outlets (a public discourse cluster rather than partisan). The chart indicates the proportion of partisan communities as a percentage of all users retweeting the party hashtags during the observation periods in the federal election campaigns in 2017 and 2021

Political hashtag debates often polarize into two or more communities. Prior research has shown that a far-right cluster led by official accounts of the AfD polarized German party hashtags (Morstatter et al. 2018; Darius and Stephany 2019). Most of the observed retweet networks of party hashtags distribute into two central clusters, (1) the AfD community and (2) another cluster containing major news outlets and politicians of other parties. For the observation periods in 2017 and 2021, the modularity-based Louvain algorithm identifies these communities, and then a qualitative content analysis assesses the 30 most retweeted accounts. This way, AfD communities and the partisan community of the other party are identified. After applying a community detection algorithm, the analysis compares the identified communities with the share of accounts of the overall network. Figure 2 displays the proportions of the pro-party partisans and contra clusters (largest community containing partisans of other parties) for the selected hashtags of major German parties. The polarization between AfD partisan and other partisan communities has increased for several hashtags, especially for the Greens and the CDU. Moreover, Table 1 summarizes the cluster proportions of the three most significant clusters in the retweet networks during the observation periods in 2017 and 2021.

The only remarkable difference is the growth of the two major clusters for #GRUENE and #CDU, caused by the AfD partisan community (contra) and their partisan community's growth in the Greens case. Except for the slight decrease in bi-polar polarization for #CSU and #FDP, there is an overall tendency of higher polarization between the two largest partisan communities, which relates to RQ1. The results indicate a higher level of bi-polar polarization for party hashtags in 2021.

### Behavior of partisan communities

**Fig. 3:**
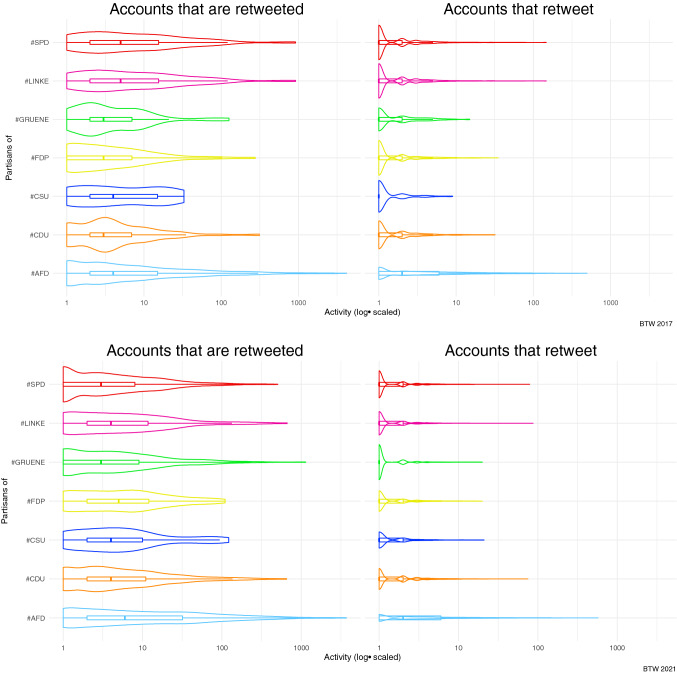
Activity of partisans from different parties contrasted between the final week of the election campaign in 2017 and 2021. The distribution of retweeting others and being retweeted is much more skewed for the AfD, where higher values are more frequent than for partisans of the other parties

While RQ1 concerned the distribution of accounts in two partisan communities, it does not account for the activity of partisan clusters. The activity of the partisan cluster is subject to RQ2, which raises the question of whether the average activity of partisan communities was higher in the 2021 elections than in the 2017 elections. Figure 3 shows the distribution of log-transformed partisan activity (accounts that retweet) and network elites (accounts that are most often retweeted by others) for partisan communities in each retweet network.

The distribution shows differences between 2017 and 2021. At first, AfD partisans are much more active than partisans of other parties. Besides, the retweets are more unequally distributed than for other communities. AfD network elites have much higher values of weighted indegree as the number of being retweeted than the elites in other party networks. Additionally, the activity of other party supporters increased notably between 2017 and 2021, especially for the AfD, FDP, and CDU, as indicated by the means in the box-candle plots in Fig. 3.

After analyzing the use of party hashtags and identifying partisan communities within the retweet networks, the analysis proceeds with analyzing the co-occurrence of these communities in the broader debate on the elections. This co-occurrence is an indicator of strategic hashtag use. Additionally, the co-occurrence of partisans from different parties indicates ideological closeness between groups (Fig. 3).

### Polarization of the broader electoral debate (RQ3a & RQ3b)

**Fig. 4:**
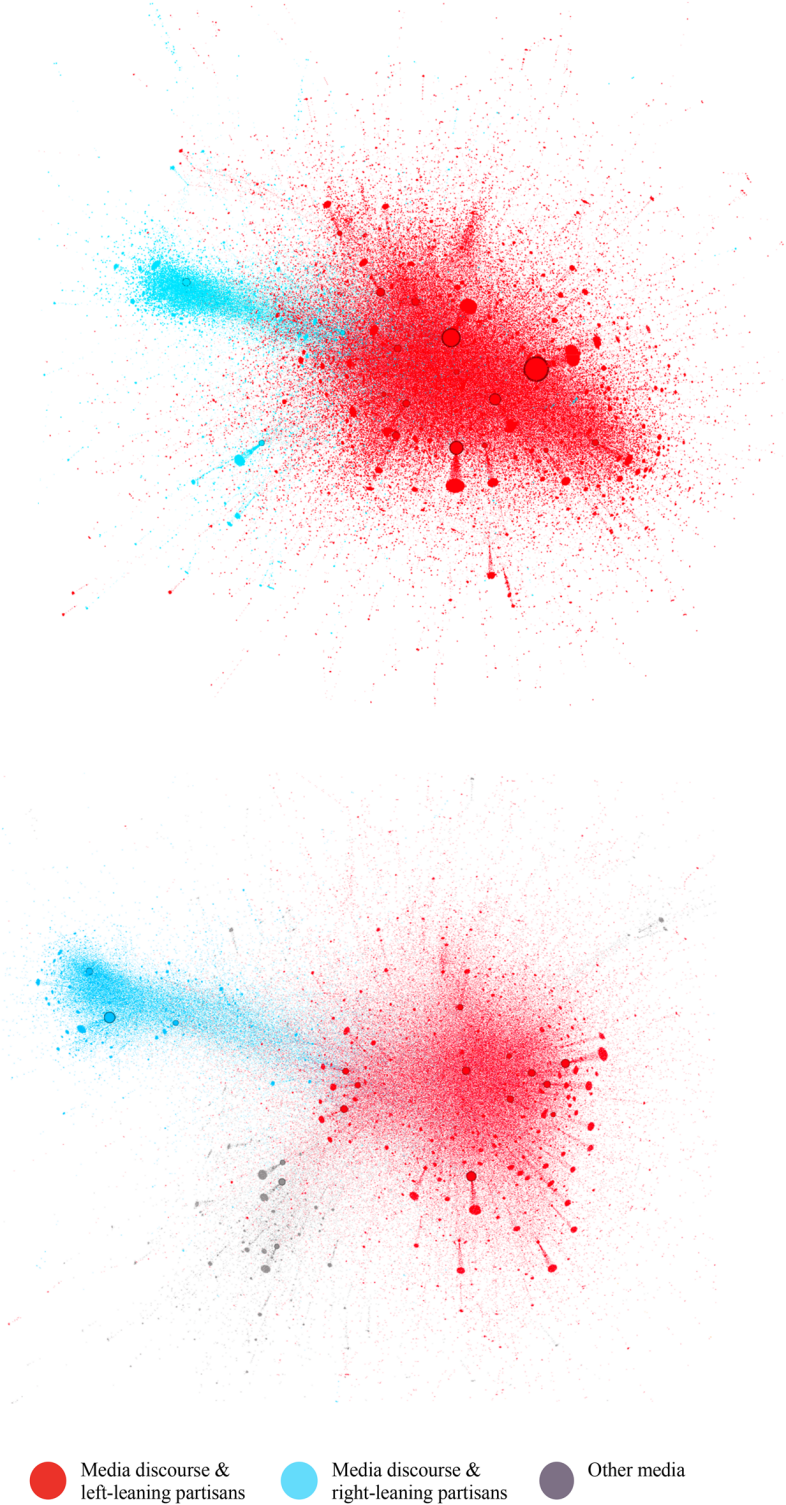
Retweet network polarization of #BTW17 (top) with 72,745 nodes and 168,239 edges and #BTW21 (bottom) with 91,789 nodes and 225,925 edges during the final week of the campaigns before the 2017 and 2021 German Federal elections. Both retweet networks are clustered and filtered to only show accounts retweeted more than 100 times during the observation period. In 2017 red cluster contains significant media outlets and all parties except the AfD (blue cluster). In 2021 red cluster contains major media outlets and left parties. However, the CDU and the main accounts of the FDP and CSU are located in the blue cluster dominated by AfD politicians

The acronym hashtags of the *Bundestagswahlen as #BTW17 and* #BTW21 are common hashtags to refer to the upcoming elections on Twitter. Figure 4 represents the retweet networks of #BTW17 (top) and #BTW21 (bottom) during the observation period. Two large clusters divide the network. The coloring indicates the community assignments of individual nodes based on the Louvain algorithm (Blondel et al. 2008; Chen et al., 2018). In contrast to earlier observation periods in 2017, official CDU, CSU, or FDP accounts are also part of the blue AfD partisan cluster in which official AfD accounts and the German tabloid BILD are the most retweeted accounts (Fig. 6). A qualitative content analysis of the 30 most retweeted accounts in Tables 2 and Table 3 shows that AfD politicians are present in the blue cluster in 2017 and 2021. In 2021, however, CDU politicians were also in the same cluster. Table 4 illustrates the changing cluster co-membership of official party accounts between 2017 and 2021. While the AfD was isolated as the only party in the blue cluster in 2017, in 2021, the AfD, CDU, CSU, and FDP are located in the blue cluster indicating a broader left–right division that the following section further investigates. The analysis proceeds by examining partisan groups retweeting political elites in the different clusters to investigate RQ4.

### The likelihood of partisan co-occurrence in the broader electoral debate (RQ4)

The analysis investigates the likelihood of co-occurrence in partisan communities and the communities in the broader discourses as an indication of strategic hashtag use. The qualitative assessment implied that the #BTW21 debate is more broadly polarized than #BTW17. The logistic regression output as the odds of co-occurrence confirms the first observation of the network analysis in Sect. (5.2) that indicated a polarization along a classical left–right party divide. Partisans of the AfD, CDU, CSU, and FDP are much more likely to occur in the right-leaning community (blue color), whereas partisans of left parties such as the Greens, SPD, and LINKE are unlikely to appear in this right-leaning cluster. In contrast to earlier studies where AfD partisans actively polarized the Twittersphere, this is a broader polarization between political ideologies in which users self-sort by their retweeting behavior. Moreover, the resulting odds for the candidate hashtag in Fig. 6 indicate a similar co-occurrence pattern.

However, it is unclear what caused these differences in partisan retweeting behaviors. Possible explanations could be, (1) political elites retweet different accounts such as media sources, (2) retweeting users/partisans retweet more closely along classical right-left divides, (3) the AfD may have become part of the conservative mainstream and is retweeted by similar users that also retweet the other parties, or the other parties have moved toward the AfD, and AfD partisans tend to be more likely to retweet CDU, CSU and FDP accounts. The following section will discuss these potential explanations and summarize the findings and limitations of the study.

## Discussion

**Fig. 5:**
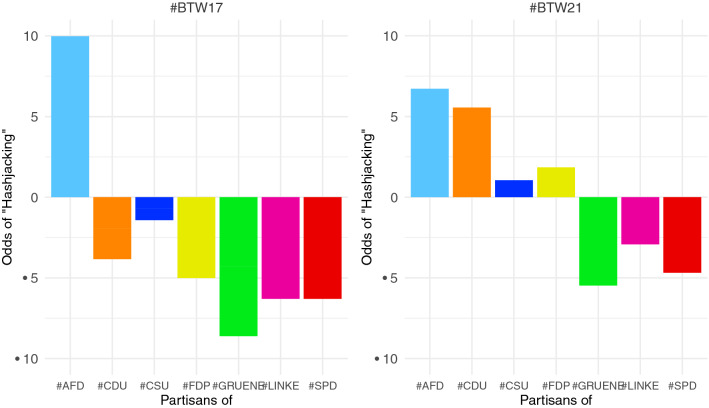
Log-likelihoods of the co-occurrence of partisans in the two major communities of the broader debate on the elections on #BTW17 in 2017 and #BTW21 in 2021. The column chart contrasts the univariate outputs of the logistics regression models for each partisan community and potentially co-occurring in the right-wing community (blue)

**Fig. 6:**
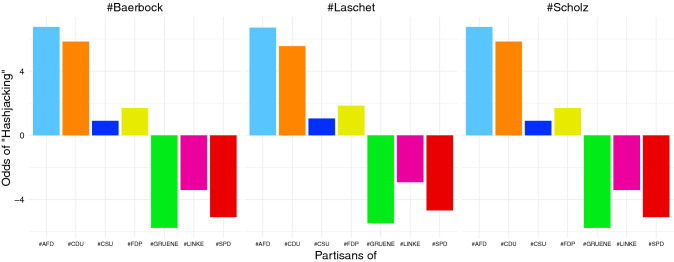
Log-likelihoods of the co-occurrence of partisans in the two major clusters/network communities of the broader debate on the elections pertaining to the chancellor candidates’ last name hashtags in 2021

This study investigates the structure of the German political party and candidate hashtags during the final week of the campaigns before the election. The analysis focuses on online political behavior such as strategic hashtag use and hashjacking, a politically motivated networked communication strategy. The term refers to a coordinated use of hashtags commonly used by or referring to a politically opposed group. While using other parties' hashtags is not per se strategic, the analyses confirm the hypotheses that partisans and politicians of the German far-right party AfD are more likely to use other parties' hashtags and appear as a partisan group in all other party hashtags displayed by the contra bars in Fig. 5. Moreover, Fig. 2 illustrates that AfD partisans show much higher activity than other, more heterogeneous groups. Understanding the mechanisms of "hashjacking" as a far-right communication strategy contributes to making sense of the overproportioned representation of far-right actors and opinions on social platforms, e.g., Twitter. During the election period, this may also result in multiplier effects regarding traditional media reporting and may therefore affect the media debate and voter decisions during the final week of the election period.

In contrast to the 2017 elections, however, AfD partisans in 2021 do not appear as an isolated group but in a joined community with CDU politicians and partisans. This joined community shows the higher likelihood of co-occurrence in Fig. 4 and shared community membership of AfD and CDU accounts that is highly apparent during the qualitative assessment of the most retweeted accounts. assessment of the most retweeted accounts in Table 2 and Table 3. The high likelihood of AfD and CDU partisan's co-occurrence in a right-wing network cluster (in #BTW21) indicates that the parties have become ideologically more similar. The following sub-sections reflect on this main finding and the study's limitations.

### Findings

The analysis showed that the Twittersphere, represented by essential political hashtags during the German Federal election, has polarized into a so-called left–right divide between party supporters. While in 2017, AfD partisans were located in an isolated cluster and effectively polarized the hashtag discourse on the elections, the 2021 #BTW21 shows a broader polarization, especially between the AfD and CDU on the one hand and Greens, SPD, and LINKE on the other side. Partisan clusters of the FDP and CSU are also in the right-wing cluster, with a lower likelihood of partisan co-occurrence as measured by the odd ratios in Fig. 4. This assessed polarization as the proportion of accounts represented in the AfD and the respective party community results from self-sorting of retweeting individuals and the content provided by political elites. For instance, in the CDU community, politicians of the party's right like Friedrich Merz or former president of the internal intelligence agency 'Verfassungschutz" Hans-Georg Maaßen, were among the most frequently retweeted. The closer location of AfD and CDU in the network structure and broader polarization may indicate a political shift of Germany's largest party, CDU. This indication of a political shift appears sensible since Friedrich Merz has become the party leader after the election and former candidate Armin Laschet stepping. Armin Laschet's resignation.

Moreover, the broader left–right polarization and higher closeness of CDU partisans and AfD partisans may indicate an increasing online polarization of social media and online news sharing and consumption that was visible in the US media system during the Trump campaign in 2016 (Benkler et al. 2018). While the AfD did not increase its vote share, it seems like the CDU has moved closer to the AfD and its online partisans or become more 'retweetable' for AfD partisans. This indicates an ideological shift and may have some forward indication since a right shift within the party was realized when Friedrich Merz became the new party leader after former party leader and chancellor candidate Armin Laschet stepped back after the elections. The findings underline a changing ideological political sphere in Germany. The study demonstrates the usefulness of network approaches for monitoring large-scale online discourses and digital political campaigning activities on social media platforms.

### Limitations of the study and methodology

The study design and methodology also come with some limitations. First, choosing hashtags as selectors also limits the analysis to the streams of debate and information (Burgess and Bruns 2012; Weller et al. 2013). However, increasing the visibility of party hashtags is a campaign goal of parties interested in increasing the frequency of messages referring to the organization and its election promises and policy plans. Concerning the assessed frequencies, the retrospective collection may not contain tweets that Twitter or the owners of the accounts, or accounts that deleted themselves or were deleted by Twitter, e.g., for automation or conflicting with the community guidelines. However, a study by Keller and Klinger (2019) indicated that automated accounts, so-called social bots, only played a limited role in the German political Twittersphere during the 2017 Federal elections.

Another limitation of the study is the focus on retweeting behavior, which leaves out quote tweets and mentions. The focus on retweeting has been chosen for two reasons. Firstly, the network structures and mechanisms differ between tweet types (Conover 2011). Secondly, the retweet networks represent the debates sufficiently since most tweets in the selected hashtag debates are retweets.

Additionally, modularity-based community detection has some limitations. Modularity values and respective cluster detection vary slightly when repeated. Due to this variation, the reproducibility of the research has its limitations. However, the edge lists of the analyzed retweet networks and Gephi output data on measures like centrality values and community memberships are published on the authors' GitHub account[1] to allow for a replication of the approach. This data allows colleagues a comparative assessment of the results, e.g., by applying various community detection algorithms to test the limits of modularity maximization for community detection (Lancichinetti and Fortunato 2009; 2011; Gates et al. 2016). The study recognizes existing research design limitations and encourages further research based on the collected data and methodological approach.

### Further research

The study finds a broader level of polarization during the election campaigns in 2021, indicating an ideological shift of the CDU. Thus, it is vital to continuously investigate the political Twitter sphere, e.g., as a potential indicator of ideological shifts in the political spectrum. Due to this visualization and analysis of political behavior, Twitter constitutes something like a political big data microscope. With regards to the findings of this study, further research needs to assess whether the level of polarization into a clear left–right divide was only a side-effect of higher politicization, activity, and, thus polarization, during the campaign period or whether it marks a lasting shift of the German political sphere. The findings align with a move toward more conservative CDU leader Merz after the elections and may have had an indicator function for ideological shifts of political parties and represent individual and party positions (Ceron 2016; Sältzer 2022).

Further research should also investigate whether membership in partisan communities varies over time and, if so, precisely for what proportion of its members. Moreover, it remains under-researched to what extent community memberships in retweet networks represent differences in political beliefs or offline political behavior. The association between online and offline political behavior can be further investigated by linking social media and survey data (Beuthner et al. 2021; Karlsen and Enjolras 2016; Stier et al. 2020; Sloan et al. 2020). Further methodological research should investigate social media data linked with panel survey data during elections to better understand to what extent the polarization of hashtag discourses represents different political alignment and a temporal perspective also opinion dynamics. Moreover, the left–right division found in the communicative behaviors could be extend by comparing language-use in the communities (Däubler and Benoit 2021). While further research should investigate the impact of the AfD and media outlets on the political sphere on- and offline, the observed broader polarization could also result from higher social media efforts by all parties. In terms of a higher activity of politicians and partisans, these efforts may result in a more explicit representation of underlying ideologies in the strategic behavior of political elites and partisans.

### Conclusions

Networked and digital campaigning has become a crucial part of election campaigning. Concerning networked digital campaigning, this study  assesses strategic communication and ideological polarization on Twitter during election campaigns. While politicians and partisans of all parties use hashtags strategically to link their tweets to broader While politicians and partisans of all parties use hashtags strategically to link their tweets to broader discourses, AfD partisans use other parties' hashtags much more frequently. This higher frequency is a sign of a purposeful hashjacking strategy that reflects the party's anti-establishment character. From the network perspective, these AfD partisans built an isolated community due to strategic retweeting behavior and ideological differences. While this was the case during the 2017 federal elections, this study finds a much broader left–right polarization of the electoral debate on Twitter during the 2021 German federal elections. On the right-leaning side of the political spectrum, partisans of the AfD, CDU, FDP, and CSU are more likely to appear within the same community, whereas politicians and partisans of the SPD, GRUENE, and LINKE are more likely to appear in the broader community with major news outlets. While the AfD intended to hashjack other party hashtags in both elections, they did not hashjack the broader electoral debate on #BTW17 and #BTW21. However, the AfD and CDU have become ideologically more similar, as indicated in a shared community membership on Twitter. This online polarization and closeness to AfD partisans resonate with or might even foreshadow a right shift of the party leadership in the months after the elections when CDU members elected Friedrich Merz as the new party leader for times in opposition. Concludingly, the study contributes to research on online political behavior during election campaigns and calls for further development of measurement methods of online discourses as measures of ideology, opinion dynamics, and political polarization.

## Appendix

See Fig. 6

See Tables 1, 2, 3 and 4

**Table 1:** Summary of collected retweet networks. Communities marked in blue are AfD partisans across all hashtag retweet networks. This high co-occurrence results from strategic hashtag use of other party hashtags by AfD politicians, which Darius and Stephany (2019) identified as hashjacking efforts

RT network	Nodes	Edges	Modularity	C1 (in %)	C2 (in %)	C3 (in %)
2021						
#BTW21	91,789	225,925	0.429	69.08	15.65	10.56
#AfD	15,572	47,947	0.385	53.95	42.44	0.2
#CSU	5,594	7,065	0.565	52.52	24.53	8.4
#CDU	18,975	34,004	0.347	72.96	21.59	0.11
#FDP	8,845	11,890	0.572	55.47	18.89	15.05
#GRÜNE	7,116	8,819	0.56	50.87	38.08	0.7
#LINKE	4,292	5,290	0.578	48.9	29.64	13.68
#SPD	10,608	15,782	0.525	52.75	37.44	0.39
#Baerbock	13,509	21,230	0.528	60.97	31.57	3.09
#Laschet	25,991	51,893	0.179	84.96	11.67	0.05
#Scholz	11,792	16,683	0.453	67.97	27.33	0.11

2017						
#BTW17	72,745	168,239	0.254	86.03	9.69	0.17
#AfD	27,713	68,781	0.497	75.10	20.57	0.10
#CSU	2,084	2,262	0.766	25.77	24.86	13.77
#CDU	4,989	7,175	0.630	36.10	33.81	8.84
#FDP	4,160	5,325	0.742	27.00	23.65	21.39
#GRÜNE	2,570	2,976	0.784	27.70	11.71	10.82
#LINKE	2,224	2,952	0.410	57.55	18.26	7.19
#SPD	6,578	9,310	0.717	28.15	25.37	15.72

**Table 2:** Top 30 most retweeted (highest weighted indegree) accounts in #BTW17 with number of unique retweeters (indegree), number of retweets (weighted indegree), community membership, and author annotation of partisanship based on qualitative content analysis of the Twitter profile

Screenname	Retweeters	Number of Retweets	Community Membership	Partisan
heuteshow	6556	9731	Red	No
tagesschau	5249	7164	Red	No
zeitonline	4527	5100	Red	No
extra3	3190	4299	Red	No
swagenknecht	1398	3010	Red	Yes
fraukepetry	1401	2821	Blue	Yes
dielinke	1033	2731	Red	Yes
spiegelonline	2071	2574	Red	No
zdfheute	2029	2565	Red	No
die_gruenen	1221	2554	Red	Yes
marspet	1731	2257	Red	No
europeelects	1200	1927	Blue	No
kiser_let	1358	1740	Red	No
raindiercks	1636	1714	Red	No
fdp	866	1610	Red	Yes
deintherapeut	1559	1592	Red	No
zdf	1297	1540	Red	No
wahlrecht_de	904	1533	Blue	No
piratenpartei	385	1385	Red	Yes
br24	950	1322	Red	No
wahlleiter_bund	1114	1278	Red	No
matthiasmeisner	702	1140	Red	No
fackjugoehte	1077	1078	Red	No
lawyerberlin	506	949	Blue	Yes
rbb24	748	880	Blue	No
krk979	450	785	Blue	Yes
mundaufmachen	421	783	Blue	Yes
poggenburgandre	466	774	Blue	Yes
niggi	609	669	Red	No

**Table 3:** Top 30 most retweeted (highest weighted indegree) accounts in #BTW21 with number of unique retweeting accounts (indegree), number of total retweets (weighted indegree), community membership, and author annotation of partisanship based on qualitative content analysis of the Twitter profile

Screenname	Number of Retweeters	Number of Retweets	Community Membership	Partisan
m_ziesmann	2436	4161	Blue	No
heuteshow	2929	3636	Red	No
hagen	2992	3163	Red	No
watch_union	1583	2949	Red	Yes
wahlen_de	1428	2658	Red	No
die_gruenen	1765	2656	Red	Yes
jumpsteady	2146	2532	Red	No
afd	1163	2522	Blue	Yes
volksverpetzer	2083	2515	Red	Yes
europeelects	1346	2468	Gray	No
wahlrecht_de	1400	2291	Red	No
dielinke	1226	2167	Red	Yes
tagesschau	1599	2120	Red	No
bild	1197	1963	Blue	Yes
campact	1335	1868	Red	No
theeconomist	1350	1806	Gray	No
abaerbock	1787	1787	Red	Yes
dwnews	1317	1753	Gray	No
afd_muenster	764	1669	Blue	Yes
der_postillon	1542	1605	Red	No
stephanschmidt	1540	1542	Red	Yes
piratenpartei	440	1475	Red	Yes
konstantinnotz	1305	1446	Red	Yes
alice_weidel	1256	1430	Blue	Yes
iblali	1221	1347	Red	No
kaffeecup	1239	1319	Red	No
lgbeutin	910	1244	Red	Yes
hartes_geld	852	1201	Red	Yes
raykanders	1059	1186	Red	No
drwaumiau	1109	1156	Red	No

**Table 4:** Cluster membership of official federal-level party accounts in 2017 and 2021. The change in cluster association indicates a shift in the political Twitter sphere

Party account	2017	2021
@afd	Blue	Blue
@CDU	Red	Blue
@CSU	Red	Blue
@fdp	Red	Blue
@Die_Gruenen	Red	Red
@dieLINKE	Red	Red
@spdde	Red	Red

## Footnotes

1 Based on the literature and searches with a social media tool.

2 While the political discourse on these platforms is public or semi-public, e.g., in large Facebook groups), the online spaces are privately owned and algorithmically governed by social platform corporations (Gillespie 2017; Katzenbach and Ulbricht 2019).

3 This representative online study by German public broadcasters ARD and ZDF summarizes results of online media use for different demographics https://www.ard-zdf-onlinestudie.de/files.

4 The differentiation between the online and offline world employed here is not a analytical, but rather a communicative distinction since digital media have become deeply entangled in our everyday offline practices.

5 Twitter started displaying content from outside the follower networks in users' home timelines to break up so-called bubbles. https://help.twitter.com/en/using-twitter/twitter-timeline.

6 https://developer.twitter.com/en/products/twitter-api/academic-research.

7 To allow reproducibility of the study, network edge lists and anonymized data on network measures is publicly accessible on the author's GitHub profile: https://github.com/philippdarius/SNAM_Darius_2022.
